# TRAF4‐Mediated LAMTOR1 Ubiquitination Promotes mTORC1 Activation and Inhibits the Inflammation‐Induced Colorectal Cancer Progression

**DOI:** 10.1002/advs.202301164

**Published:** 2024-01-16

**Authors:** Linlin Zhao, Ni Gao, Xiaoping Peng, Lei Chen, Tong Meng, Cong Jiang, Jiali Jin, Jiawen Zhang, Qiuhui Duan, Hongling Tian, Linjun Weng, Xinbo Wang, Xiao Tan, Yaxu Li, Huanlong Qin, Jian Yuan, Xin Ge, Lu Deng, Ping Wang

**Affiliations:** ^1^ Tongji University Cancer Center Shanghai Tenth People's Hospital Shanghai Frontiers Science Center of Nanocatalytic Medicine School of Medicine Tongji University Shanghai 200092 P. R. China; ^2^ Department of Orthopedics Shanghai General Hospital School of Medicine Shanghai Jiaotong University Shanghai 200940 P. R. China; ^3^ Department of Gastrointestinal Surgery Shanghai Tenth People's Hospital Tongji University Shanghai 200092 P. R. China; ^4^ Research Institute of Intestinal Diseases Tongji University School of Medicine Shanghai 200092 P. R. China; ^5^ Department of Biochemistry and Molecular Biology Tongji University School of Medicine Shanghai 200092 P. R. China; ^6^ Research Center for Translational Medicine Shanghai East Hospital Tongji University School of Medicine Shanghai 200120 P. R. China; ^7^ College of Animal Science and Technology Northwest A&F University Yangling Shaanxi 712100 P. R. China

**Keywords:** LAMTOR1, mTORC1, Rag GTPase, TRAF4, ubiquitination

## Abstract

Mechanistic target of rapamycin complex 1 (mTORC1) is a conserved serine/threonine kinase that integrates various environmental signals to regulate cell growth and metabolism. mTORC1 activation requires tethering to lysosomes by the Ragulator‐Rag complex. However, the dynamic regulation of the interaction between Ragulator and Rag guanosine triphosphatase (GTPase) remains unclear. In this study, that LAMTOR1, an essential component of Ragulator, is dynamically ubiquitinated depending on amino acid abundance is reported. It is found that the E3 ligase TRAF4 directly interacts with LAMTOR1 and catalyzes the K63‐linked polyubiquitination of LAMTOR1 at K151. Ubiquitination of LAMTOR1 by TRAF4 promoted its binding to Rag GTPases and enhanced mTORC1 activation, K151R knock‐in or TRAF4 knock‐out blocks amino acid‐induced mTORC1 activation and accelerates the development of inflammation‐induced colon cancer. This study revealed that TRAF4‐mediated LAMTOR1 ubiquitination is a regulatory mechanism for mTORC1 activation and provides a therapeutic target for diseases involving mTORC1 dysregulation.

## Graphical abstract

A model demonstrating the ubiquitination of LAMTOR1 in response to amino acids availability. Upon amino acids stimulation, the E3 ligase TRAF4 bind to LAMTOR1 and promote the K63‐linked ubiquitination of LAMTOR1 at K151, which increased its binding with Rag GTPases and the activation of mTORC1.

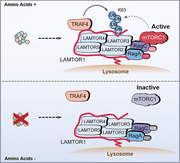

## 1 Introduction

Mechanistic target of rapamycin complex 1 (mTORC1) is an essential nutrient‐sensing kinase that orchestrates both intracellular and extracellular signals to regulate a wide range of cellular functions.^[^ ^1^ ^]^ mTORC1 activity is tightly controlled by the status of nutrients, such as amino acids, glucose, and lipids.^[^ ^2^ ^]^ Once activated, mTORC1 potentiates protein and lipid biosynthesis, inhibits autophagy, and promotes cell growth by phosphorylating a panel of protein substrates such as ribosomal protein S6 kinase 1 (S6K1), eukaryotic initiation factor 4E‐binding protein 1 (4E‐BP1), transcription factor EB (TFEB), and Unc‐51‐like autophagy activating kinase 1 (ULK1).^[^ ^3^ ^]^ The dysregulation of mTORC1 is associated with various diseases, including cancer, metabolic diseases, and developmental disorders.^[^ ^4^ ^]^

The activation of mTORC1 relies on two small guanosine triphosphatases (GTPases): Rag^[^ ^5^ ^]^ and Rheb.^[^ ^6^ ^]^ Four Rag proteins have been identified: RagA, RagB, RagC, and RagD. RagA/B interacts with RagC/D to form a heterodimeric complex (RagA/B bound to RagC/D) that is essential for tethering mTORC1 from the cytosol to the lysosomal surface. The GTP‐bound RagA/B and GDP‐bound RagC/D heterodimeric complex is the activated form that recruits mTORC1 to the lysosomes.^[^ ^5^ ^]^ The Rag complex is activated by interactions with Ragulator on lysosomes and is inhibited by the GATOR1 complex.^[^ ^7^, ^8^, ^9^ ^]^ mTORC1 is recruited to lysosomes and is activated by direct interaction with Rheb, which is activated by growth factors.^[^ ^10^ ^]^

The Ragulator complex is essential for mTORC1 activation via functioning as a guanine nucleotide exchange factor (GEF) for RagC/D to convert RagC/D from an inactive GTP‐bound form to an active GDP‐bound form.^[^ ^11^ ^]^ Ragulator is a pentamer containing five proteins: LAMTOR1, LAMTOR2, LAMTOR3, LAMTOR4, and LAMTOR5,^[^ ^7^, ^12^ ^]^ and form two heterodimers: LAMTOR2–LAMTOR3 and LAMTOR4–LAMTOR5. LAMTOR1 acts as a rope linking heterodimers and is essential for the formation of Ragulator pentamer and mTORC1 activation.^[^ ^13^, ^14^, ^15^ ^]^ Lysosomal localization of LAMTOR1 mediated by myristoylation and palmitoylation is essential for Ragulator‐Rag complex to activate mTORC1,^[^ ^16^, ^17^ ^]^ and the binding of Ragulator to RAG GTPases is dynamically regulated;^[^ ^18^ ^]^ however, the underlying mechanism remains unclear.

Accumulating evidence has shown that ubiquitination, an important post‐translational modification that usually conjugates the small polypeptide ubiquitin (Ub) to the amino group of the lysine residues of substrates,^[^ ^19^ ^]^ plays a critical role in mTORC1 activation. For example, the E3 ligase TRAF6 regulates lysosomal translocation of mTOR by catalyzing K63 ubiquitination of mTOR.^[^ ^20^ ^]^ Polyubiquitination of mLST8 determines mTORC1 formation.^[^ ^21^ ^]^ DEPTOR is ubiquitinated and degraded by β‐TrCP1^[^ ^22^ ^]^ and OTUB1.^[^ ^23^ ^]^ In addition, the K63 linkage polyubiquitination of RagA mediated by RNF152 or SKP2 regulates the RagA‐mTORC1 pathway.^[^ ^24^, ^25^ ^]^ Moreover, RHEB activity is also controlled by ubiquitination.^[^ ^26^ ^]^ A recent study indicated that the ubiquitination of Sestrin2 modulates the activation of mTORC1.^[^ ^27^ ^]^ The LAMTOR1 protein has also been reported to be degraded by E3 ligase UBE3A‐mediated ubiquitination at K60, K103, and K104.^[^ ^28^ ^]^ Deubiquitination of LAMTOR1 at K20 by USP32 promotes mTORC1 activation by regulating its interaction with v‐ATPase.^[^ ^29^ ^]^ However, whether the activity of Ragulator toward Rag GTPase is regulated by ubiquitination remains unclear.

In the current study, we found that LAMTOR1 was modified by K63‐linked polyubiquitination, which was correlated with amino acid availability. TRAF4 is a specific E3 ligase that targets LAMTOR1 K151 for Lys63‐linked polyubiquitination. Functionally, the ubiquitination of LAMTOR1 regulates the interaction between Ragulator and Rag GTPases and enhances the GEF activity of Ragulator toward RagC/D, which in turn promotes the activation of mTORC1 and affects the progression of inflammation‐induced colorectal cancer (CRC). Thus, we identified TRAF4‐mediated LAMTOR1 ubiquitination as a potential mechanism for the dynamic interaction between Ragulator and Rag.

## 2 Results

### 2.1 Amino Acid‐Induced K63‐Linked Polyubiquitination of LAMTOR1

**Figure 1:**
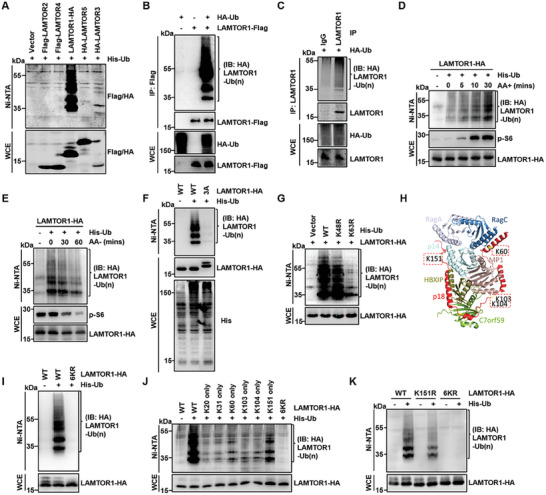
Amino acid‐induced K63‐linked polyubiquitination of LAMTOR1 at K151. A) LAMTOR1 was ubiquitinated but no other components of Ragulator. The ubiquitination levels of Ragulator components were analyzed in HEK293T cell after the transfection of different plasmids. The ubiquitinated proteins were pulled down under the denature condition by Ni‐NTA agarose beads and were detected by western blotting. B) The ubiquitination of LAMTOR1 was detected by immunoprecipitation (IP) assay, and IP was performed from HEK293T cell transfected with HA‐Ub and LAMTOR1‐Flag plasmids. C) Ubiquitination of endogenous LAMTOR1 was detected in HEK293T cell with transfection of HA‐Ub plasmid by immunoprecipitation (IP) assay. D) The ubiquitination of LAMTOR1 was regulated by amino acids signaling. HEK293T cell transfected with the His‐Ub and LAMTOR1‐HA plasmids was starved in DMEM without amino acids for 60 min and re‐stimulated with amino acids for 5, 10 or 30 min. E) HEK293T cell transfected with the His‐Ub and LAMTOR1‐HA plasmids was starved in DMEM without amino acids for 30 or 60 min. F) Ubiquitination of LAMTOR1‐3A was detected in HEK293T cell transfected with LAMTOR1‐HA or its mutation. G) The ubiquitination type of LAMTOR1 is K63‐linked poly‐ubiquitination. H) The structure model of Ragulator. LAMTOR1 has six lysine residues which could be potentially ubiquitinated. I) All mutations of LAMTOR1 six lysine residues (LAMTOR1‐6KR) completely abolished LAMTOR1 ubiquitination. J) LAMTOR1‐K151 only mutant displayed higher ubiquitination comparing to other LAMTOR1‐K only mutations. K) LAMTOR1‐K151R displayed lower ubiquitinationl than LAMTOR1‐WT while no ubiquitination was detected in LAMTOR1‐6KR.

As a key regulator of the mTORC1 pathway, the dynamic regulation of the activity of the Ragulator complex toward Rag GTPases in response to amino acid stimulation remains unknown. As Ragulator regulates mTORC1 activation via direct interaction with Rags,^[^ ^18^ ^]^ we examined whether Ragulator is modified by ubiquitination, an important mechanism that regulates protein interactions.^[^ ^30^ ^]^ To this end, we examined the ubiquitination of each component of Ragulator and found that LAMTOR1, but not the other components, was significantly ubiquitinated in HEK293T cells (**Figure** **1**A and B). We confirmed that the endogenous LAMTOR1 could also be ubiquitinated (Figure 1C; Figure S1A, Supporting Information). Next, we examined whether nutrient status affected LAMTOR1 ubiquitination and found that amino acids, but not glucose or EGF, promote the ubiquitination of LAMTOR1 (Figure 1D; Figure S1B, and S1C, Supporting Information). In contrast, the depletion of amino acids significantly inhibited LAMTOR1 ubiquitination (Figure 1E). Surprisingly, we found that blocking mTORC1 activity with rapamycin inhibited LAMTOR1 ubiquitination (Figure S1D, Supporting Information). The knockout (KO) of NPRL2, a key component of GATOR1, increased LAMTOR1 ubiquitination with or without amino acids (Figure S1E, Supporting Information). These data prompted us to hypothesize that amino acids may induce LAMTOR1 ubiquitination via a positive feedback mechanism and play a role in mTORC1 activation.

LAMTOR1 binds to the lysosomal membrane through myristoylation and palmitoylation of the N‐terminal amino acid residues 2, 3, and 4.^[^ ^16^, ^17^ ^]^ We examined whether lysosomal localization of LAMTOR1 was involved in its ubiquitination. To this end, we mutated N‐terminal amino acid residues 2, 3, and 4 to Ala (LAMTOR1‐3A) and assessed its ubiquitination. Our data showed that the ubiquitination of LAMTOR1‐3A was markedly reduced (Figure 1F), suggesting that lysosomal localization is required for ubiquitination.

Ubiquitination is repeated to form various types of ubiquitin chains that differentially control the fate of substrates.^[^ ^31^ ^]^ We then examined the linkage specificity of LAMTOR1 for ubiquitination and found that LAMTOR1 ubiquitination was decreased in cells expressing a ubiquitin mutant (Ub‐K63R), in which the 63 lysine residue was replaced with arginine residue, but not in other ubiquitin mutants (Figure 1G; Figure S1F, Supporting Information). These data suggested that LAMTOR1 underwent K63‐linked polyubiquitination.

### 2.2 K151 as the Major Ubiquitination Site of LAMTOR1

Ubiquitin is usually attached to the amino groups of lysine residues on substrates. To map the ubiquitination sites of LAMTOR1, we mutated six lysine residues (K20, K31, K60, K103, K104, and K151) (Figure 1H) and found that the mutation of all lysine residues completely abolished LAMTOR1 ubiquitination (Figure 1I), indicating that the ubiquitin chains were attached to the lysine residues. One lysine‐containing mutant with only 151, but not the other lysine residues, showed strong ubiquitination (Figure 1J). Consistently, the replacement of K151 with R strongly reduced LAMTOR1 ubiquitination (Figure 1K). Importantly, similar to the wild type (WT), the ubiquitination of the K151 single mutant was dynamically regulated by amino acid stimulation (Figure S1G, Supporting Information). We also noticed that K151 is conserved in mammals, rodents, birds, amphibians, and fish LAMTOR1 sequences (Figure S1H, Supporting Information), suggesting that K151 ubiquitination may be involved in sensing amino acids across species.

### 2.3 TRAF4 as an E3 Ligase of LAMTOR1

**Figure 2:**
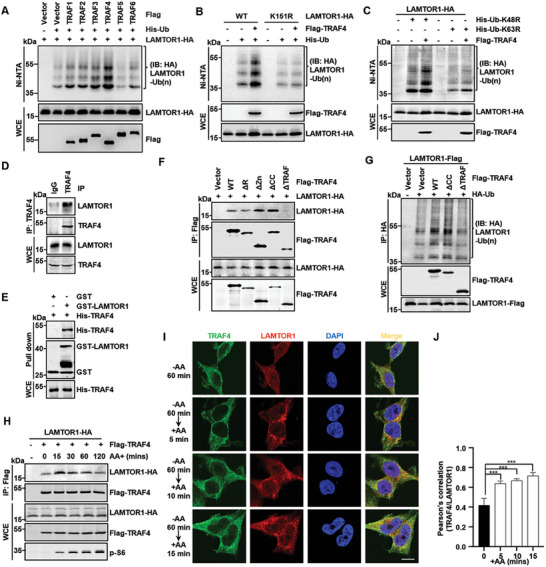
TRAF4 as an E3 ligase of LAMTOR1. A) Screening the E3 ubiquitin ligase of LAMTOR1. The ubiquitination of LAMTOR1 was analyzed in HEK293T cell, which was transfected with different E3, His‐Ub and LAMTOR1‐HA plasmids. The ubiquitinated proteins were pulled down under denature condition by Ni‐NTA‐agarose beads and detected via western blotting. B) The ubiquitination of LAMTOR1‐WT, but not LAMTOR1‐K151R mutant was promoted by TRAF4. C) TRAF4 promotes Lys63 linked ubiquitin chains of LAMTOR1. D) The binding of endogenous LAMTOR1 and TRAF4. Endogenous TRAF4 was immunoprecipitated from HEK293T cell by rabbit TRAF4 polyclonal antibody or control rabbit IgG antibody. Coimmunoprecipitated endogenous LAMTOR1 was analyzed via western blotting. E) His‐TRAF4 could directly interact with GST‐LAMTOR1 which was detected by GST pull‐down assay. F) TRAF4 interacted with LAMTOR1 is dependent on its C‐terminal TRAF domain. G) TRAF4 with the loss of TRAF domain could not promote LAMTOR1 ubiquitination. H) The interaction between TRAF4 and LAMTOR1 is regulated by amino acid signaling. HEK293T cell was transfected with the Flag‐TRAF4 and LAMTOR1‐HA plasmids. Then the cell was starved with amino acids deficient DMEM for 60 min and re‐stimulated with amino acids for 0, 15, 30, 60, 120 min. I) Immunofluorescent staining for endogenous TRAF4 (green) and endogenous LAMTOR1 (red). HEK293T cell was starved of amino acids for 60 min and re‐stimulated with amino acids for 5, 10, 15 min. Scale bar, 10 µm. J) Pearson's correlation quantifies endogenous TRAF4/LAMTOR1 colocalization, mean ± SD (n ≥ 5), two‐tailed Student's t test, ***p < 0.001.

E3 ligase is responsible for substrate selection and is important for the formation of ubiquitin chains formation.^[^ ^31^ ^]^ Since LAMTOR1 is localized to the lysosome and our data suggest that the lysosomal localization of LAMTOR1 is important for its ubiquitination. Moreover, previous studies have shown that the TRAF N‐terminus can promote the recruitment of TRAFs to the membrane.^[^ ^32^ ^]^ These prompted us to examine membrane‐localized E3 ligases TRAFs may be involved in LAMTOR1 ubiquitination. Thus, we screened E3 ligase TRAFs and identified that TRAF4, but not other E3, significantly promoted LAMTOR1 ubiquitination (**Figure** **2**A). Ubiquitination of LAMTOR1 by TRAF4 was confirmed in HEK293T cells (Figure S2A, Supporting Information), and our in vitro ubiquitination assay showed that TRAF4 could directly promote K63‐linked polyubiquitination of LAMTOR1 (Figure S2B, Supporting Information). TRAF4‐C18A, an E3 ligase non‐active mutant,^[^ ^33^ ^]^ failed to induce LAMTOR1 ubiquitination (Figure S2C, Supporting Information), indicating that TRAF4‐mediated LAMTOR1 ubiquitination is dependent on its E3 ligase activity. LAMTOR1 has been shown to be degraded by the E3 ligase UBE3A in a ubiquitin proteasome‐dependent manner in the hippocampal neurons of mice.^[^ ^28^ ^]^ Our data showed that the expression of TRAF4, but not UBE3A, significantly promotes the ubiquitination of LAMTOR1 in HEK293T cells (Figure S2D, Supporting Information). These data suggest that LAMTOR1 ubiquitination is regulated by different E3 ligases in a cell type‐dependent manner.

We examined whether TRAF4 ubiquitinated LAMTOR1 at K151 and found that TRAF4 failed to ubiquitinate the LAMTOR1‐K151R mutant (Figure 2B) but promoted the ubiquitination of LAMTOR1‐K151 only mutant (Figure S2E, Supporting Information). TRAF4‐mediated ubiquitination of LAMTOR1 was dramatically reduced upon the ectopic expression of Ub‐K63R (Figure 2C). Thus, we concluded that TRAF4 is the E3 ligase responsible for K63‐linked ubiquitination of LAMTOR1 at K151.

Next, we evaluated the interaction between TRAF4 and LAMTOR1 using a co‐immunoprecipitation (co‐IP) assay and found that LAMTOR1, but not the other components of Ragulator, strongly interacted with TRAF4 (Figure 2D; Figure S2F–I, Supporting Information). Our in vitro pull‐down assay showed that TRAF4 directly interacted with LAMTOR1 (Figure 2E). To identify the TRAF4 domains required for the interaction with LAMTOR1, we constructed a series of truncated TRAF4 mutations (Figure S2J, Supporting Information). Our data showed that absence of the C‐terminal TRAF domain (TRAF4‐ΔTRAF) completely abrogated its binding to LAMTOR1 (Figure 2F) and failed to induce LAMTOR1 ubiquitination (Figure 2G). These data indicated that TRAF4 interacts with LAMTOR1 through its TRAF domain to regulate LAMTOR1 ubiquitination.

Next, we examined whether the interaction between TRAF4 and LAMTOR1 was regulated by amino acid stimulation. Our data showed that the interaction between TRAF4 and LAMTOR1 was increased and then decreased upon stimulation with amino acids (Figure 2H), which is consistent with the amino acid‐induced ubiquitination of LAMTOR1. Interestingly, we found that TRAF4 co‐localized with the lysosomal marker LAMP2 (Figure S2K, Supporting Information). Accordingly, the colocalization of both exogenous and endogenous TRAF4 with LAMTOR1 was enhanced upon amino acid stimulation (Figure 2I,J; Figure S2L and S2M, Supporting Information). Together, these data suggest that amino acid induces the ubiquitination of LAMTOR1 by regulating the interaction between TRAF4 and LAMTOR1 on lysosomes.

### 2.4 Regulation of the Interaction Between Rag GTPases and LAMTOR1 by TRAF4‐Mediated Ubiquitination of LAMTOR1

**Figure 3:**
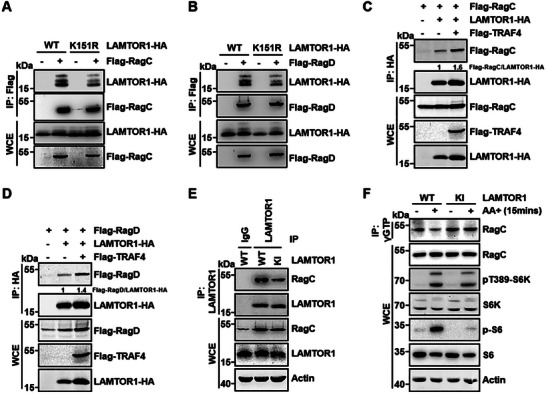
The ubiquitination of LAMTOR1 promotes its interaction with Rag GTPases. A,B) The interactions between LAMTOR1‐K151R and RagC or RagD were decreased comparing to LAMTOR1‐WT. Flag‐RagC or Flag‐RagD was co‐transfected with LAMTOR1‐HA WT and K151R mutant into HEK293T cell. Then the cell was lysed and RagC was immunoprecipitated by anti‐Flag antibody. Co‐immunoprecipitated LAMTOR1‐HA WT and K151R mutation were detected by anti‐HA antibody. C,D) TRAF4 promoted the interactions between LAMTOR1 and RagC or RagD. LAMTOR1‐HA and co‐precipitated RagC/D band intensities in the IP were separately performed with a normalization analysis via Image J. E) The binding of endogenous WT or K151R LAMTOR1 with RagC in HEK293T WT or KI cell lines. F) LAMTOR1 K151R mutation affects the activation of RagC using g‐amino‐hexyl‐GTP beads in HEK293T cell. GTP‐bound RagC was detected via western blotting.

Lys63‐linked polyubiquitin chains are non‐degradable signals that often orchestrate the formation of protein complexes.^[^ ^34^ ^]^ Our data showed that TRAF4 overexpression had little effect on the abundance of LAMTOR1 (Figure S3A, Supporting Information), indicating that TRAF4 does not target LAMTOR1 for degradation. The most important function of Ragulator is to recruit Rag GTPases to the lysosomal surface and regulate their activity.^[^ ^11^, ^12^ ^]^ Previous studies have shown that the highly conserved C‐terminus of LAMTOR1, which contains two prominent charged and hydrophobic clusters (151‐KEELVV‐156), is required to recruit Rags.^[^ ^13^ ^]^ However, how these residues contribute to the function of Ragulator remains unknown. Our data showing that LAMTOR1 K151 is ubiquitinated by TRAF4 prompted us to examine whether LAMTOR1 ubiquitination affects its binding to Rag GTPases. We found that LAMTOR1‐6KR, a ubiquitination‐resistant mutant, significantly reduced the binding of LAMTOR1 to RagC (Figure S3B, Supporting Information). Similarly, LAMTOR1‐K151R also displayed decreased binding affinity for Rag GTPases (**Figure** **3**A,B; Figure S3C–H, Supporting Information). Moreover, amino acid stimulation promoted the binding of RagC to LAMTOR1‐WT, but not to K151R (Figure S3I, Supporting Information). TRAF4 overexpression promoted the binding of LAMTOR1 to RagC/D (Figure 3C and D). These data indicate that the ubiquitination of LAMTOR1 by TRAF4 promotes the binding of LAMTOR1 and RagC/D.

To examine whether ubiquitination of endogenous LAMOTR1 at K151 affects Ragulator function, we generated a *LAMTOR1 K151R* knock‐in (KI) HEK293T cell line using CRISPR‐Cas9. *K151R* KI did not affect the protein levels of Ragulator components and Rag GTPases (Figure S3J, Supporting Information), nor the interaction between LAMTOR1 and other Ragulator components (Figure S3K, Supporting Information). We further confirmed that the endogenous binding of LAMTOR1‐K151R to RagC was significantly lower than that of LAMTOR1‐WT (Figure 3E). We then examined whether the LAMTOR1 K151R mutation affected RagC activation and found that this mutation significantly blocked RagC activation in response to amino acid stimulation (Figure 3F). Taken together, these data indicated that LAMTOR1 ubiquitination at K151 promoted the interaction between Ragulator and Rag GTPases and enhanced their activation.

### 2.5 LAMTOR1 Ubiquitination Promoted mTORC1 Activation

**Figure 4:**
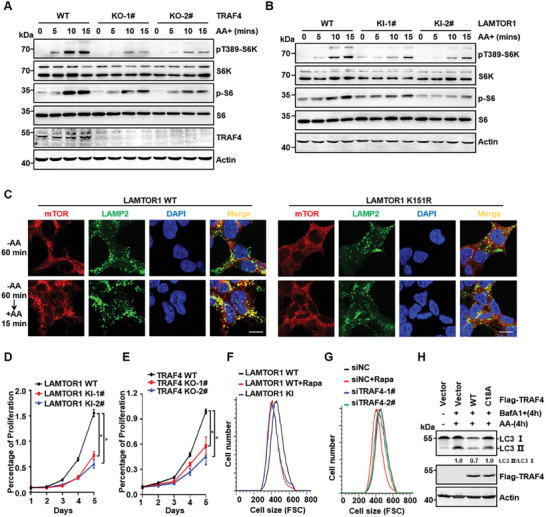
LAMTOR1 ubiquitination promotes mTORC1 activation. A,B) The activation of mTORC1 was decreased in TRAF4 deficient or LAMTOR1 K151R knock‐in HEK293T cell lines. C) The mutation of LAMTOR1 at K151 site negatively regulated the localization of mTOR to the lysosome in LAMTOR1 K151R knock‐in HEK293T cell. Scale bar, 10 µm. D,E) The cell proliferation was analyzed using an MTT assay in the LAMTOR1 K151R knock‐in and TRAF4 knockout HEK293T cell lines. The cell proliferation rate was normalized to control group. Data were analyzed by Student's t‐test. *p*< 0.05 was considered statistically significant. F) FACS analysis was performed to determine cell size in LAMTOR1 K151R knock‐in HEK293T cell lines. G) HEK293T cells were transfected with control or TRAF4 siRNAs (siTRAF4), and FACS analysis was performed. H) TRAF4‐WT rather than TRAF4‐C18A mutation reduced the autophagy in HEK293T cell. FCS, forward cell scatter; siNC, nonspecific control siRNA.

Next, we examined whether TRAF4‐mediated LAMTOR1 ubiquitination was involved in mTORC1 activation. Our data showed that depletion of TRAF4, but not the E3 ligase TRAF2, dramatically decreased amino acid‐induced S6K1 phosphorylation (Figure S4A and S4B, Supporting Information). This result was confirmed by the CRISPR‐Cas9‐based (KO) of TRAF4 (*TRAF4^−/−^*) in HEK293T, and we also confirmed in CRC cell lines, such as SW480, RKO, SW620 and HT29 (**Figure** **4**A; Figure S4C–F, Supporting Information). Consistently, TRAF4 deficiency in MEFs markedly inhibited the amino acid‐induced mTORC1 activation (Figure S5A and S5B, Supporting Information). In contrast, TRAF4 knockdown had minor effects on EGF‐ and glucose‐induced mTORC1 activation (Figure S5C and S5D, Supporting Information). Taken together, these data indicated that TRAF4 is a positive regulator of amino acid‐induced mTORC1 activation.

Next, we examined whether the effect of TRAF4 on mTORC1 activation was mediated by the TRAF4‐mediated ubiquitination of LAMTOR1 at K151. *LAMTOR1‐K151R* KI HEK293T cells were treated with different amino acids at different time points. We found that the LAMTOR1‐K151R mutation reduced amino acid‐induced S6K1 phosphorylation, as well as the phosphorylation of S6, a target of S6K (Figure 4B). We also generated *LAMTOR1‐K151R* KI mice to confirm this result (Figure S6A and S6B, Supporting Information). *LAMTOR1‐K151R* KI mice were born at expected Mendelian ratios (data not shown), indicating that LAMTOR1 K151 ubiquitination is not required for embryonic development. To investigate whether LAMTOR1 K151R was involved in mTORC1 regulation, we derived MEFs from *LAMTOR1‐K151R* KI embryos and found that amino acid‐induced mTORC1 activation was significantly decreased in K151R KI MEF (Figure S6A–C, Supporting Information). Furthermore, amino acid‐induced mTORC1 translocation was largely reduced in K151R KI HEK293T cells compared to WT cells (Figure 4C; Figure S6D, Supporting Information). TRAF4 deficiency in LAMTOR1 K151R cells did not significantly affect the activity of the mTORC1 signaling pathway (Figure S6E, Supporting Information), suggesting that TRAF4 regulates the mTORC1 signaling pathway through ubiquitination of LAMTOR1. Together, these findings demonstrate that LAMTOR1 ubiquitination at K151 is essential for amino acid‐induced activation of mTORC1.

mTORC1 is also involved in cell proliferation and growth.^[^ ^4^ ^]^ Therefore, we examined whether TRAF4‐mediated LAMTOR1 ubiquitination affects cell proliferation. Our data showed that either the LAMTOR1 K151R KI or the TRAF4 KO resulted in a significant decrease in the proliferation rate (Figure 4D and E). We also measured cell size and found that LAMTOR1 K151R KI and TRAF4 KO cells were smaller than control cells (Figure 4F and G), confirming the important role of TRAF4‐mediated LAMTOR1 ubiquitination in the cellular function of mTORC1. Amino acid deprivation can induce autophagy via inhibition of mTORC1 activity.^[^ ^35^ ^]^ Our data showed that autophagy‐induced by amino acid starvation was markedly inhibited by the overexpression of the WT, but not by the non‐active mutant TRAF4 (Figure 4H). Overall, these data illustrate that LAMTOR1 ubiquitination by TRAF4 controls cell proliferation, size, and autophagy. Interestingly, the morphology of *LAMTOR1 K151R* and LAMTOR1‐KO cells significantly changed to a cluster growth phenotype (Figure S6F, Supporting Information), which has been previously reported as a typical phenotype of LAMTOR1 KO cells.^[^ ^36^ ^]^ These data suggest that TRAF4‐mediated ubiquitination of LAMTOR1 at K151 is involved in the regulation of cell morphology.

### 2.6 Positive Correlation of TRAF4 Expression with mTORC1 Activation in Cancer

**Figure 5:**
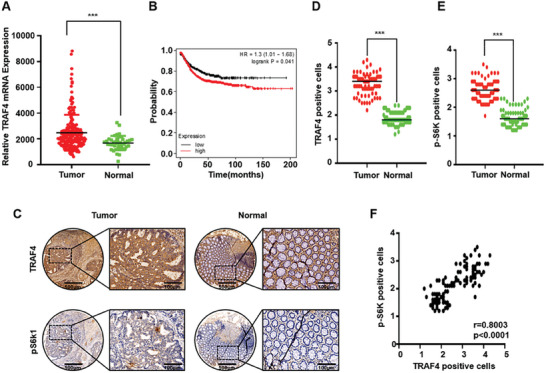
The positive correlation of TRAF4 expression with mTORC1 activation in cancer. A) TRAF4 mRNA was significantly increased in colorectal cancer from TCGA database. Tumor (*n* = 192) and Normal (*n* = 41). Data were analyzed by Student's t‐test. ***p < 0.001. B) TCGA database showed that the high expression of TRAF4 was positively correlated with the poor prognosis of colorectal cancer patients. C) Representative images of TRAF4 and p‐S6K immunohistochemistry staining in colorectal cancer or normal tissues specimens. D–F) The expressions of TRAF4 and p‐S6K in colorectal cancer were significantly higher comparing to normal tissues, and the expression trends of these two proteins were positively correlated. Data were analyzed by Student's t‐test. ***p < 0.001. TCGA, The Cancer Genome Atlas.

As the activation of mTORC1 is correlated with tumorigenesis, we analyzed the expression levels of TRAF4 by searching the TCGA database. We found that TRAF4 was upregulated in colon cancer and that high expression levels of TRAF4 were positively correlated with poor prognosis (**Figure** **5**A and B). To examine whether TRAF4 expression was correlated with mTORC1 activity, we performed tissue microarray analysis of TRAF4 expression and mTORC1 activation in colon cancer samples (Figure 5C). Our data showed that the increased TRAF4 protein expression levels were positively correlated with elevated mTORC1 activity (monitored by the level of p‐S6K) in colon cancer tissues (Figure 5D–F). These data suggest that TRAF4 plays an important role in colon tumor growth by regulating mTORC1 activation.

### 2.7 Inhibition of Inflammation‐Induced Cancer Progress by LAMTOR1 Ubiquitination

**Figure 6:**
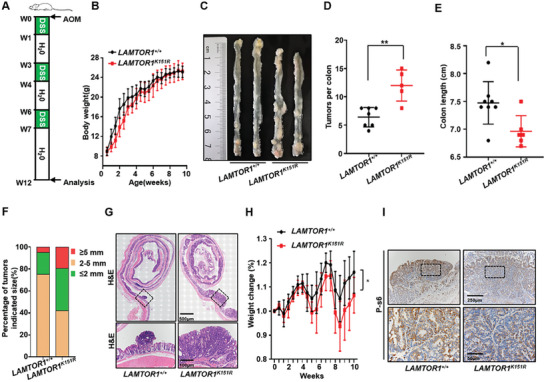
Inhibition of inflammation induced cancer progressing by LAMTOR1 ubiquitination. A) A scheme of AOM/DSS colorectal cancer model. AOM, azoxymethane; DSS, dextran sodium sulfate. B) Growth curve of *LAMTOR1^+/+^* (*n* = 7) and *LAMTOR1^K151R^* (*n* = 7) mice in 10 weeks. C) Representative image of colon tumors in mice on the 12 weeks after injection of AOM. D‐F) Number of polyps; colorectal length; percentage of colorectal tumors size from *LAMTOR1^+/+^* and *LAMTOR1^K151R^* mice treated with AOM/DSS. Data were analyzed by Student's t‐test. P < 0.05 was considered statistically significant. *p < 0.05, **p < 0.01. G) HE staining of mouse colorectal cancer samples. H) Body weight changes of *LAMTOR1^+/+^* (*n*  =  10) and *LAMTOR1^K151R^* (*n*  =  9) mice in AOM/DSS colon cancer model. I) Representative immunohistochemical p‐s6 staining of AOM/DSS colon cancer samples.

To examine whether TRAF4‐mediated LAMTOR1 ubiquitination has any function in vivo in an inflammation‐induced CRC mouse model that has been shown to be inhibited by the mTORC1 pathway,^[^ ^37^ ^]^ we examined the effect of TRAF4 KO (*TRAF4 ^−/−^*) mice using an azoxymethane (AOM)/dextran sodium sulfate (DSS)‐induced colon cancer model (**Figure** **6**A). Our data revealed that TRAF4 KO markedly increased the number of tumor colonies and shortened the colon length induced by AOM/DSS inflammation‐induced CRC (Figure S7A–C, Supporting Information), but had little effect on mouse body weight (Figure S7D, Supporting Information). Furthermore, we examined whether LAMTOR1 ubiquitination at K151 is involved in inflammation‐induced CRC using *LAMTOR1 K151R* KI mice. We found that the body weight of mice was not significantly affected over a ten‐week period after birth by *LAMTOR1 K151R* KI (Figure 6B). However, *LAMTOR1 K151R* KI mice (Figure 6C) showed a higher number of tumor colonies (Figure 6D), shorter colon length (Figure 6E), and larger tumor size (Figure 6F and G) than in WT mice in an AOM/DSS inflammation‐induced CRC. Consistently, the body weight in *LAMTOR1 K151R* KI mice was lost more than in WT mice (Figure 6H). In addition, the lethality of *LAMTOR1 K151R* KI male mice was higher than that of WT mice during AOM/DSS induction (Figure S7E, Supporting Information). Importantly, our data showed that p‐S6 was reduced, Ki67 was increased in tumors from *LAMTOR1 K151R* KI mice compared to that in WT mice (Figure 6I; Figure S7F, Supporting Information), which is consistent with the findings of Marta Brandt et al.^[^ ^37^ ^]^ that mTORC1 inactivation promotes AOM/DSS‐induced CRC. Taken together, these data indicated that TRAF4‐mediated LAMTOR1 ubiquitination inhibited inflammation‐induced CRC development.

## 3 Discussion

**Figure 7:**
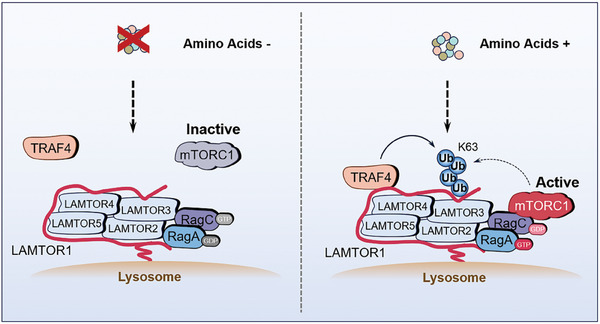
Model of the ubiquitination of LAMTOR1 mediated by amino acids. A) A model regarding the ubiquitination of LAMTOR1 in response to amino acids availability. Upon amino acids stimulation, the E3 ligase TRAF4 binds to LAMTOR1 and promotes the K63‐linked ubiquitination of LAMTOR1 at K151, which increases its binding to Rag GTPases and the activation of mTORC1.

In the current study, we propose a mechanistic model for amino acid‐induced mTORC1 activation, in which TRAF4‐catalyzed ubiquitination of LAMTOR1 enhances mTORC1 activation by promoting the interaction between Ragulator and Rag GTPase (**Figure** **7**). We found that amino acid stimulation promoted the K63‐linked ubiquitination of LAMTOR1 at K151. Blocking LAMTOR1 ubiquitination decreases its binding to RAG GTPases and mTORC1 activation. LAMTOR1 K151 ubiquitination is mainly catalyzed by the E3 ligase TRAF4. Interestingly, TRAF4‐mediated LAMTOR1 ubiquitination promotes mTORC1 activation and inhibits inflammation‐induced CRC. Overall, our findings indicate that TRAF4‐mediated K63‐linked polyubiquitination of LAMTOR1 is an important mechanism that controls mTORC1 activation and plays a surprising role in inflammation‐induced CRC development (Figure 7).

As an important regulatory protein that activates mTORC1 via amino acid signaling, the Ragulator complex is mainly located on the lysosomal surface and interacts with and activates Rag GTPases.^[^ ^7^, ^12^ ^]^ LAMTOR1 anchors the Ragulator complex to the surface of lysosomes through its N‐terminal structure^[^ ^15^ ^]^ and is vital for the function of the Ragulator complex. Our data indicated that blocking myristoylation and palmitoylation of LAMTOR1 blocked LAMTOR1 ubiquitination by TRAF4. As myristoylation and palmitoylation of LAMTOR1 are not only required for its lysosomal localization but also the formation of the Ragulator complex, we cannot exclude the possibility that a mechanism other than lysosome localization is required for LAMTOR1 ubiquitination. Moreover, LAMTOR1 ubiquitination was blocked by rapamycin treatment and promoted by NPRL2 KO, suggesting that mTORC1 may also regulate LAMTOR1 ubiquitination through a feedback loop. As previous studies showed that the activity of TRAF4 may be regulated by phosphorylation,^[^ ^38^ ^]^ it is possible that phosphorylation or other post‐translational modifications may be involved in this feedback regulation. Taken together, our results reveal a novel regulatory mechanism for mTORC1 activation via TRAF4‐mediated LAMTROR1 ubiquitination.

The current study reported that amino acid abundance promoted K63‐linked ubiquitination of LAMTOR1 at the conserved K151. Functionally, we confirmed that K151 plays an essential role in controlling the Ragulator/Rag interaction and mTORC1 activation, both in vitro and in vivo. Notably, our results are also consistent with structural studies showing that C‐terminal residues containing K151 are crucial for Ragulator function.^[^ ^13^, ^14^, ^15^, ^39^ ^]^ Together, our results indicate that LAMTOR1 ubiquitination plays a critical role in controlling Ragulator function.

In addition to interacting with Rag GTPases, Ragulator has been shown to interact with other proteins, such as v‐ATPase, AXIN,^[^ ^40^ ^]^ NLPR3,^[^ ^41^ ^]^ RIPK1, and caspase‐8.^[^ ^42^, ^43^ ^]^ Consistently, various functions of the Ragulator complex have been reported, such as the activation of the NLRP3 inflammasome, the function of AMPK, MAPKs, and pyroptosis.^[^ ^41^, ^43^ ^]^ Moreover, we also observed that K151 is involved in the regulation of cell morphology by Ragulator. Therefore, it is worthwhile to examine whether LAMTOR1 ubiquitination affects other Ragulator functions.

mTORC1 plays various roles in different types of CRC.^[^ ^44^ ^]^ For example, CRC with APC mutations shows increased mTORC1 activity, which is associated with enhanced tumor growth.^[^ ^45^ ^]^ On the other hand, in inflammation‐induced CRC, inactivation of mTORC1 leads to increased chromosomal instability and impairs regeneration of intestinal crypts, triggers interleukin‐6‐associated reparative inflammation, and induces crypt hyper‐proliferation, wound healing, and CRC.^[^ ^37^ ^]^ It has proved that blocking IL‐6 signaling can reduce inflammation‐induced colorectal cancer. This means that inflammatory cells in the tumor microenvironment also play an important role in colorectal tumor occurrence.^[^ ^37^ ^]^ Although we did not confirm this mechanism in our model, it may explain why we obtained opposite phenotypes in vitro (Figure 4D) and in vivo (Figure S6F, Supporting Information). Analysis of CRC samples from mice with AOM/DSS inflammation‐induced CRC showed that *K151R* KI mice had shorter colons, a higher number of larger CRC tumors, and a significant reduction in lifespan, indicating that LAMTOR1 ubiquitination has an inhibitory effect on inflammation‐CRC. Although our data showed that mTORC1 activation was reduced in *K151R* KI mice, we cannot exclude other functions of LAMTOR1 such as the NLRP3 inflammasome, which may also contribute to inflammation‐induced CRC.

TRAF4 has been identified as a key molecule in a variety of ontogenetic processes, particularly in the nervous system in mouse and fly models.^[^ ^46^ ^]^ Numerous studies have shown that TRAF4 expression is upregulated in human cancers.^[^ ^47^ ^]^ However, the role of TRAF4 in cancer, especially inflammation‐induced cancer, remains unknown. Through TCGA data analysis, we found that TRAF4 is highly expressed in human CRC and that high expression of TRAF4 is positively correlated with increased mTORC1 activity in colon cancer samples. Although we could not determine the types of CRC, our data suggest that TRAF4 promotes colon cancer in humans via mTORC1 activation. Surprisingly, using a mouse model, we found that TRAF4 inhibits inflammation‐induced colon cancer progression, possibly via the ubiquitination of LAMTOR1. These data suggested that TRAF4 is involved in cancer progression in a context‐dependent manner.

## 4 Conclusion

In conclusion, we propose a mechanistic model in which Ragulator‐mediated mTORC1 activation is regulated by the TRAF4‐catalyzed ubiquitination of LAMTOR1 at K151, which regulates the development of inflammation‐induced colon cancer. Therefore, LAMTOR1 ubiquitination and TRAF4 may serve as useful therapeutic targets for diseases involving mTORC1 dysregulation.

## 5 Experimental Section

### Cell Culture and Transfection

All cell lines were received from the Chinese Academy of Sciences Committee Type Culture Collection Cell Bank (Shanghai, China) and authenticated by the cell banks with short tandem repeat analysis. Both human embryonic kidney 293T (HEK293T), mouse embryonic fibroblast cells (MEF) and Human Colorectal Carcinoma cell line (HT29, RKO) were cultured in DMEM medium supplemented with 10% heat‐inactivated fetal bovine serum (FBS, Gibco) and 1% penicillin‐streptomycin (PS) in a 37 °C humidified incubator with 5% CO_2_. Human Colorectal Carcinoma cell line (SW480, SW620) was cultured in RPMI 1640 medium supplemented with 10% FBS and 1% PS at 37 °C humidified incubator with 5% CO_2_. HEK293T cells were transfected with calcium phosphate‐DNA co‐precipitation method or PEI (Polysciences) separately.

### Plasmids and RNA Interference (RNAi)

The His‐ubiquitin (His‐Ub) and its mutants were described previously.^[^ ^48^ ^]^ LAMTOR1 and its mutants were cloned into pcDNA3.1 vector with Flag‐ or HA‐tag on the C terminus. TRAF4 and its mutants were cloned into pcDNA3.1 vector with Flag‐ or HA‐tag on the N terminus. RagA, B, C, and D were cloned into pcDNA3.1 vector with Flag‐tag on the N terminus. GST‐tagged LAMTOR1 was cloned into pGEX‐4T‐2. His‐tagged TRAF4 was cloned into pET28a vector. All constructs were confirmed via DNA sequencing.

siRNA oligonucleotides were transfected using Lipofectamine 2000 (Invitrogen). siRNA transfection of cells were performed according to the manufacturer's instructions. The sequences of siRNAs against TRAF4 were indicated as follows:

siRNA‐TRAF4#1 (5′‐CCUGCACCUACUGCACUAATT‐3′)

siRNA‐TRAF4#2 (5′‐GCACUAAGGAGUUCGUCUUTT‐3′)

### Reagents and Antibodies

Anti‐Flag, anti‐HA, and secondary antibodies were purchased from Sigma (St. Louis, MO). The anti‐GFP (B‐2 sc‐9996), Ubiquitin (P4D1 sc‐8017), LAMP2 (H4B4 sc‐18822) antibodies were obtained from Santa Cruz Biotechnology (Santa Cruz, CA). The antibodies against LAMTOR1 (3783S), LAMTOR2 (3783S), LAMTOR3 (3783S), LAMTOR4 (3783S), LAMTOR5 (3783S), pS6K T389 (9234S/L), pS6 (4858S, 1:3000), mTOR (2983S), S6K (9202S), S6 (2217S, 1:3000), RagA (4357S), RagB (D18F3), RagC (D18F3) and RagD (D18F3) were purchased from CST. The anti‐TRAF4 (66755‐1‐lg) was purchased from Proteintech. The anti‐LC3II (L7543) was purchased from Sigma. The anti‐beta Actin (S0312, 1:3000) was purchased from DiagBio. Tag antibodies were used at 1:3000 dilution rate for immunoblotting. All other antibodies were used for immunoblotting at a concentration of 1:1000. The MG132 (S2619) was from Selleck. Ni‐NTA Agarose (30 210) was from QIAGENE. Besides, DMEM (Amino‐acid‐free) was purchased from Genetimes Technology while amino acid (50X) was purchased from Gibco.

### Immunoblot and Immunoprecipitation

For immunoblotting, cells were washed once with ice‐cold PBS and lysed in SDS‐PAGE loading buffer (1× with β‐Mercaptoethanol) and boiled at 100 °C for 15 min, resolved by 8%–15% SDS‐PAGE and analyzed by immunoblotting. Immunoprecipitation and Western Blotting were depicted as previously described. Take 6‐well plate as an example, the HEK293T cells at 90% confluence in a 6‐well plate and each well was lysed in 500 µl CHAPS lysis buffer (10 mм β‐glycerophosphate, 0.3% CHAPS, 1 mм EDTA, 40 mм HEPES, pH 7.4, 120 mм NaCl, and a cocktail of proteinase inhibitors). After sonication for 10 min, the soluble part of cell lysates was centrifugated at 12 000 rpm in a frozen microcentrifuge for 15 min. Then the cell lysates were centrifuged to discard the cell debris and incubated with 7 µl HA (Abmart) or 5 µl M2 beads (Sigma) for 2 h. After three times washing with CHAPS buffer, the beads were boiled and resolved using SDS‐PAGE gel electrophoreses and analyzed by western blotting. The result can be measured and analyzed by Odyssey system or used by the electrochemiluminescence (ECL) imaging system (Tanon). Image J was used for the quantitative analyses.

### Viral Preparation and Infection

To prepare retrovirus for knockout experiment, HEK293T cell at 60% confluence in a 6‐well plate was transduced by lentiCRISPR v2 vector and retrovirus packaging vectors psPAX2 and pMD2G using the PEI method. The retrovirus packaging system is 0.2 µg pMD2G, 0.4 µg psPAX2, 1 µg sgRNA, and 11 µl PEI. Medium containing virus was collected after 48 h of the transfection and cleared with 0.45 mm filter. The HEK293T cell was cultured with the collected viral supernatant in the presence of polybrene. Infected cell was selected with 1 µg mL^−1^ puromycin after 48 h post‐infection and propagated for further analysis.

### Protein Purification

His‐TRAF4, His‐HA‐Ub, His‐Ubch6, and GST‐LAMTOR1 were purified from *E. coli*. The plasmids were delivered into BL21 (DE3) *E. coli*. Samples (4 ml) were cultured at 37 °C at beginning. Next day, took 400 µl *E. coli* contained culture into 250 ml fresh bacterial culture medium and shaked at 37 °C, 220 rpm until the OD600 reached 0.6. Then, 0.2 mм IPTG (isopropyl‐b‐D‐thiogalactoside) was added to medium, and the cultures were shaked at 16 °C, 220 rpm for 18 h. Recombinant proteins were purified from collected pellet. *E. coli* pellet was resuspended in lysis buffer (20 mм Tris‐HCl, pH 8.0, 500 mм NaCl, protease inhibitor cocktail) and sonicated for 30 min (35% amplitude) on ice. Then, the lysates were centrifuged at 8 000 rpm for 30 min, 200ul Ni‐NTA beads was added into the lysates and incubated for 1–2 h at 4 °C. After that the beads were washed by wash buffer (lysis buffer containing 20 mм imidazole) for 3 times. After that, the protein was eluted with elution buffer (20 mм Tris‐HCl, 250 mм imidazole, 0.15 м NaCl, pH8.0). GST‐tagged protein was washed by wash buffer (lysis buffer) without imidazole and eluted with GSH elution buffer (50 mм Tris‐HCl, 10 mм reduced glutathione, pH 8.0).

### GST‐Pull Down Assay

His‐TRAF4 protein was purified from *E. coli* and incubated with 10 µg of purified GST or GST‐LAMTOR1 protein. The GST proteins were purified using GSTSep Glutathione Agarose Resin (Yeasen, 20507ES10), reacted in 800 µl pull‐down buffer (25 mм Tris‐HCl, pH 7.4‐7.6, 150 mм NaCl, and a cocktail of proteinase inhibitors) and incubated 4–5 h at 4 °C. The bound TRAF4 was detected using western blotting.

### Ubiquitination Assay

When performing the ubiquitination assay, HEK293T cell at 40% confluence in 6‐well plate was co‐transfected with His‐Ub (1.5 µg). After 24 h, the transfected cells were lysed using 900 µl metamorphic Buffer A (0.1 м Na_2_HPO_4_/NaH_2_PO_4_, 6 м guanidine‐HCl, 10 mм imidazole, pH 8.0). Then the ubiquitinated proteins were refined using 15 µl Ni‐NTA beads (the beads was pre‐washed three times with Buffer A) and rotated 12 h at room temperature. Then washed with Buffer B (0.01 м Tris‐HCl, pH 8.0, 10 mм β‐mercaptoethanol, 8 м urea, 0.1 м Na_2_HPO_4_/NaH_2_PO_4_, pH 8.0), Buffer C (8 м urea, 0.1 м Na_2_HPO_4_/NaH_2_PO_4_, pH 6.3, 10 mм β‐mercaptoethanol, 0.01 м Tris‐HCl, pH 6.3) with 0.2% Triton X‐100, and Buffer C. After the above steps, beads was incubated with 40 µl elution buffer (0.72 м β‐mercaptoethanol, 30% glycerol, 200 mм imidazole, 0.15 м Tris‐HCl, pH 6.7, 5% SDS) at room temperature rotated for 30 min and then suspended in 20 µl SDS‐PAGE sample buffer (4× with β‐Mercaptoethanol). The samples were heated at 100 °C for 15 min and can be analyzed using immunoblotting.

### In Vitro Ubiquitination Assay

GST‐LAMTOR1 protein was generated in *E. coli*. Flag‐TRAF4 plasmid was transfected into HEK293T cell in 10‐cm dish at a 50% cell density. After 24 h, the cell lysate was collected and incubated with 15–20 µl anti‐FLAG beads for 2 h, and then Flag‐TRAF4 was eluted from beads using 3 X FLAG peptide (P9801, Beyotime). The purified proteins were incubated with ubiquition activating enzyme E1(UBE1 50 ng) (Sino Biological), UbcH6 (200 ng), Ubiquition (10 µg), Ubiquition‐K63R (10 µg), 500 ng Flag‐TRAF4 and 200 ng GST‐LAMTOR1 at 37 °C for 3 h in reaction buffer (50 mм Tris‐HCl, pH 7.5, 5 mм MgCl_2_, 1 mм DTT, 2 mм ATP). Then the reaction was terminated by the addition of SDS‐PAGE sample buffer. The samples were boiled at 100 °C for 5 min and analyzed by immunoblotting.

### Amino Acid Starvation and Re‐stimulation

Cells were grown in a 12‐well plate and cultured to 40%–50% confluence. All cells were washed once with PBS and incubated in 500 µl amino acid‐free DMEM for 60 min, and then restimulated by amino acid (50X, Gibco) for indicated time. The final concentration of amino acids in the media was parallel in DMEM. The medium was removed on ice and then added 150 µl SDS‐PAGE sample buffer.

### GTP‐Binding Assay

For binding of Rags to GTP‐Agarose beads, LAMTOR1 WT or K151R mutant HEK293T cells were seeded into 10‐cm dish. After amino acid starvation and restimulation, the cells were harvested and suspended in 700 µl binding buffer (20 mм HEPES pH 8.0, 150 nм NaCl, 10 mм MgCl_2_, and a cocktail of proteinase inhibitors) and lysed using three freeze thaw cycles. The cell lysates were centrifuged at 12 000 rpm and the supernatants were incubated with 100 µl of GTP‐Agarose suspension (G9768, Sigma) overnight with rotation at 4 °C. The beads were washed three times in binding buffer, suspended in 40 µl SDS‐PAGE sample buffer and analyzed by western blotting.

### MTT Assay

Cell proliferation was detected by MTT assay. HEK293T cell line with LAMTOR1‐K151R knockin and TRAF4 knockout were seeded into 96‐well plate at a density of 700 cells well^−1^. After 24, 48, 72, 96, and 120 h, 200 µl of MTT (5 mg ml^−1^) was mixed into each well (protect from the light). The samples were incubated for 4 h at 37 °C and then in medium with 200 µl of dimethyl sulfoxide (DMSO) at room temperature for 10 min. Three independent wells were analyzed at 490 nm wavelength using spectrophotometer.

### Cell Size Analysis

To detect the cell size, HEK293T cell line with LAMTOR1 K151R knockin or HEK293T cells were transfected with TRAF4 siRNAs. The cells were cultured on 12 well with moderated density and subcultured into 10 cm‐dish after 24 h transfection. Then the cells were cultured at 37 °C for 48 h. The medium should be changed every 24 h. Cells were harvested and subjected to FACS analysis to measure the cell size.

### Immunofluorescence

1 × 10^5^ HEK293T cells were plated on fibronectin‐coated glass coverslips in 24‐well plates. 24 h later, cells were starved with amino acid‐free DMEM for 60 min and restimulated by amino acids. Then the cells were rinsed twice with PBS, and fixed for 30 min with 4% paraformaldehyde at room temperature. Then the fixed cells were washed three times with PBST (0.05% Tween‐20 in PBS), permeabilized with 0.1% Triton X‐100 for 30 min, and rinsed three times with PBST. The coverslips were blocked for 1 h with blocking buffer (1% BSA in PBS) and incubated with primary antibody in blocking buffer overnight at 4 °C. The primary antibodies mTOR (CST#2983S, 1:200), LAMP‐2 (Santa Cruz#sc‐18822, 1:100), HA‐Tag (Santa Cruz#sc‐18822, 1:500), Flag‐Tag (Proteintech#20543‐1‐AP, 1:500), TRAF4 (Proteintech#66755‐1‐Ig, 1:200), LAMTOR1 (CST##8975, 1:200) were used. Next day the coverslips were rinsed three times with blocking buffer and incubated with secondary antibodies for 1 h at room temperature in the dark, followed by tyramide signal amplification. The secondary antibodies Alexa Fluor 488‐conjugated Anti‐Mouse IgG (Jackson#111‐545‐044, 1:800) and Alexa Fluor 594‐conjugated Anti‐Rabbit IgG (Jackson#111‐585‐003, 1:800) were used. Glass coverslips were mounted on Mowiol and examined with a Zeiss LSM 510 Meta confocal system.

### Human Tissue Microarray and Immunohistochemistry (IHC) Analysis

Ethics of research projects involving human subject was approved by the Ethics Committee of Shanghai Tenth People's Hospital with approval number SHSY‐IEC‐KY −4.0/18‐120/01. The tissue microarray (TMA) of colon cancer samples was obtained from Shanghai Tenth People's Hospital. Colon cancer TMA was composed by 75 patient samples with histologically diagnosed colon cancer. The control groups (73 samples) were their paratumoral colonic epithelium. The TMA was immunohistochemically stained using a kit from Dako (Copenhagen). Antibodies were presented in TRAF4 (Proteintech#66755‐1‐lg), pS6K (CST#9202) and Ki67 (Servicebio#GB111141‐100). Nuclei were counterstained with hematoxylin (Vector Laboratories). Immunostaining on each slide was assessed by two experienced pathologists with histochemistry score (H‐score). H‐score = Σpi (i + 1) where i represents the intensity score and pi represents the percentage of cells with that intensity.

### Generation of LAMTOR1 K151R KI and TRAF4 KO Mice

Experimental animal ethics was approved by the Experimental Animal Ethics Committee of Shanghai Tenth People's Hospital with approval number SHDSYY‐2018‐3316. *LAMTOR1 K151R* knock‐in and TRAF4 knockout mice were generated using CRISPR‐Cas9 methods. Briefly, guide RNA (gRNA) expression vectors were constructed into pGS3‐T7‐gRNA. pGS3‐T7‐gRNA vector and Cas9‐encoding plasmids were linearized by DraI and NotI respectively. Linearized templates were in vitro transcribed through run‐off reactions by T7 RNA polymerase using the In vitro Transcription T7 Kit (Takara) and Sp6 mMESSAGE mMACHINE Kit (Ambion) respectively. TE solution containing 25 ng µl^−1^ of gRNA and 50 ng µl^−1^ of Cas9 mRNA was injected into the cytoplasm of one‐cell stage embryos. T7E1 mismatch sensitive assay was used to identify founders. To confirm the modifications in founders, PCR products from each founder were cloned using TA cloning kit (Takara) following the manufacturer's instructions. PCR was used to identify the genotype of offsprings from mixed mice intercrossing.

### Generation of MEF Cell

Briefly, Mice homozygous for LAMTOR1 K151R or TRAF4 were intercrossed, the pregnant female mice were sacrificed at day 13 post‐coitum, dissected out the individual embryos, and removed any extra embryonic tissues. Then the embryo was dispersed using scissor, the dispersed tissues were trypsin treated at 37 °C for 30 mins, inactivated trypsin by adding DMEM medium (contains ten percent fetal bovine serum). Cells were isolated by centrifugation at 1 000 rpm in a microcentrifuge for 5 min at room temperature, then the cells were resuspended in DMEM medium and were plated on 10 cm dishes.

### AOM/DSS Inflammation‐Induced Colon Cancer Model

AOM/DSS treatment was performed as previously described.^[^ ^49^ ^]^ Briefly, at 10 weeks of age mice were injected intraperitoneally with 10 mg k^−1^ g AOM (Sigma) and treated with 1.2% DSS (MP Biomedicals) in drinking water for 7 days, then drank normal water for 2 weeks. DSS treatment was repeated every 3 weeks. Repeated 3 times until 9 weeks, then the mice were fed normally. Mice were sacrificed ≈100 days after AOM injection.

### Statistical Analysis

All the quantification analysis was performed using GraphPad Prism 8. Data were shown as mean ± SD, Statistical significance was evaluated using two‐tailed Student's t‐tests for comparison of two mean values, as indicated in figure legends. *p < 0.05, **p < 0.01, ***p < 0.001, ns, not significant. No statistical methods were used to predetermine sample size.

## Conflict of Interest

The authors declare no conflict of interest.

## Supporting information

Supporting Information

## Data Availability

The data that support the findings of this study are available from the corresponding author upon reasonable request.
