# Supplementary material for: TRAF4‐Mediated LAMTOR1 Ubiquitination Promotes mTORC1 Activation and Inhibits the Inflammation‐Induced Colorectal Cancer Progression
Source: Adv Sci (Weinh). 2024 Jan 16;11(12):2301164. doi: 10.1002/advs.202301164 (PMC10966530; doi:10.1002/advs.202301164)
Supplement: Supplementary file 1 — Supporting Information [file ADVS-11-2301164-s001.pdf]

ADVANCED SCIENCE
Open Access

Supporting Information

for *Adv. Sci.*, DOI 10.1002/advs.202301164

TRAF4-Mediated LAMTOR1 Ubiquitination Promotes mTORC1 Activation and Inhibits the Inflammation-Induced Colorectal Cancer Progression

Linlin Zhao, Ni Gao, Xiaoping Peng, Lei Chen, Tong Meng, Cong Jiang, Jiali Jin, Jiawen Zhang, Qiuhui Duan, Hongling Tian, Linjun Weng, Xinbo Wang, Xiao Tan, Yaxu Li, Huanlong Qin, Jian Yuan, Xin Ge, Lu Deng and Ping Wang**

Supporting Information

TRAF4-mediated LAMTOR1 ubiquitination promotes mTORC1 activation and inhibits the inflammation-induced colorectal cancer progression

Linlin Zhao, Ni Gao, Xiaoping Peng, Lei Chen, Tong Meng, Cong Jiang, Jiali Jin, Jiawen Zhang, Qiuhui Duan, Hongling Tian, Linjun Weng, Xinbo Wang, Xiao Tan, Yaxu Li, Huanlong Qin, Jian Yuan, Xin Ge, Lu Deng, Ping Wang**

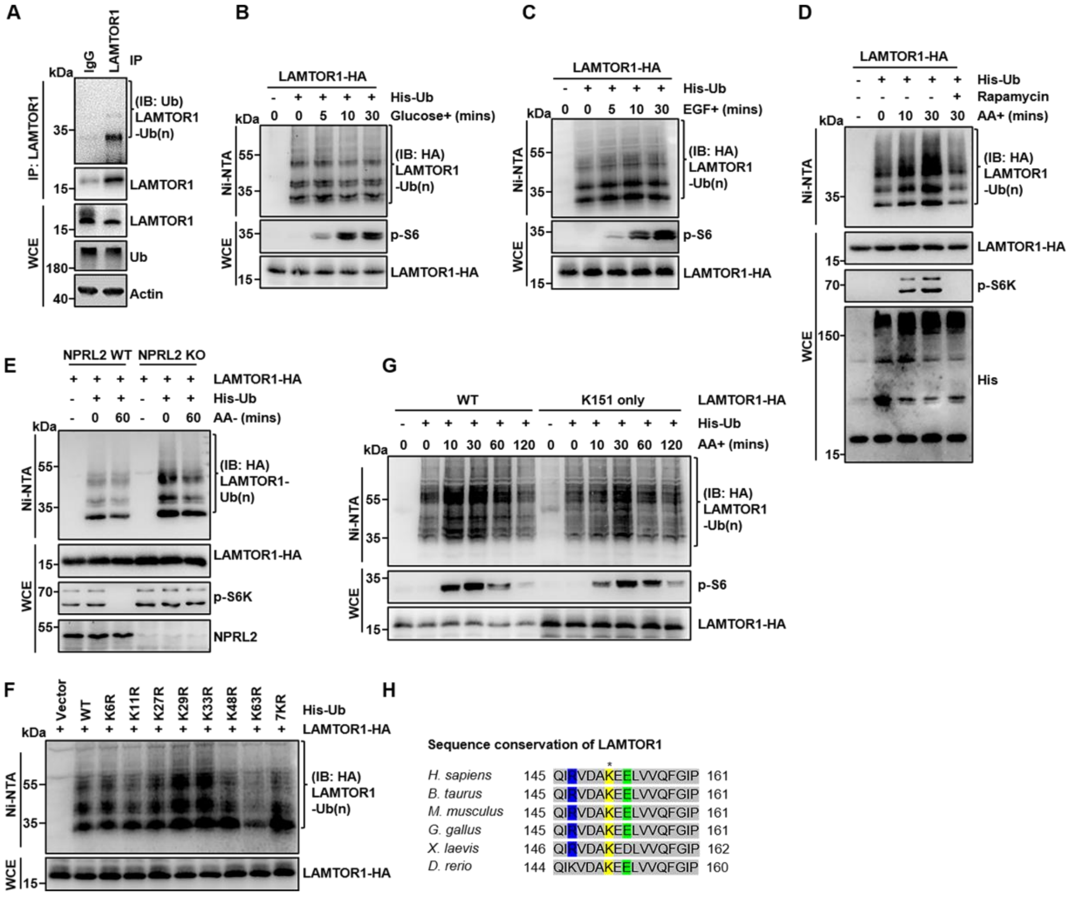

Figure S1. Amino acid-induced K63-linked polyubiquitination of LAMTOR1 at K151

A) Ubiquitination of endogenous LAMTOR1 was detected in HEK293T cells. The ubiquitination of LAMTOR1 was detected by immunoprecipitation (IP) assay. B-C) The glucose and EGF signaling had no effect on the ubiquitination of LAMTOR1. D) The ubiquitination of LAMTOR1 was regulated by amino acids signaling and suppressed by Rapamycin. HEK293T cells transfected with the His-Ub and LAMTOR1-HA plasmids were starved of amino acids for 60 min and re-stimulated with amino acids for 10min, 30min or 30min with Rapamycin. Rapamycin was incubated for 30min with 20nM. E) NPRL2 KO increase the ubquitination of LAMTOR1 in HEK293T. F) The ubiquitination type of LAMTOR1 is K63-linked poly-ubiquitination. G) The ubiquitination of LAMTOR1-K151 only was regulated by amino acids signaling, which was similar to LAMTOR1-WT. H) The K151 of LAMTOR1 is conserved in mammals, rodents, birds, amphibians and fish sequences.

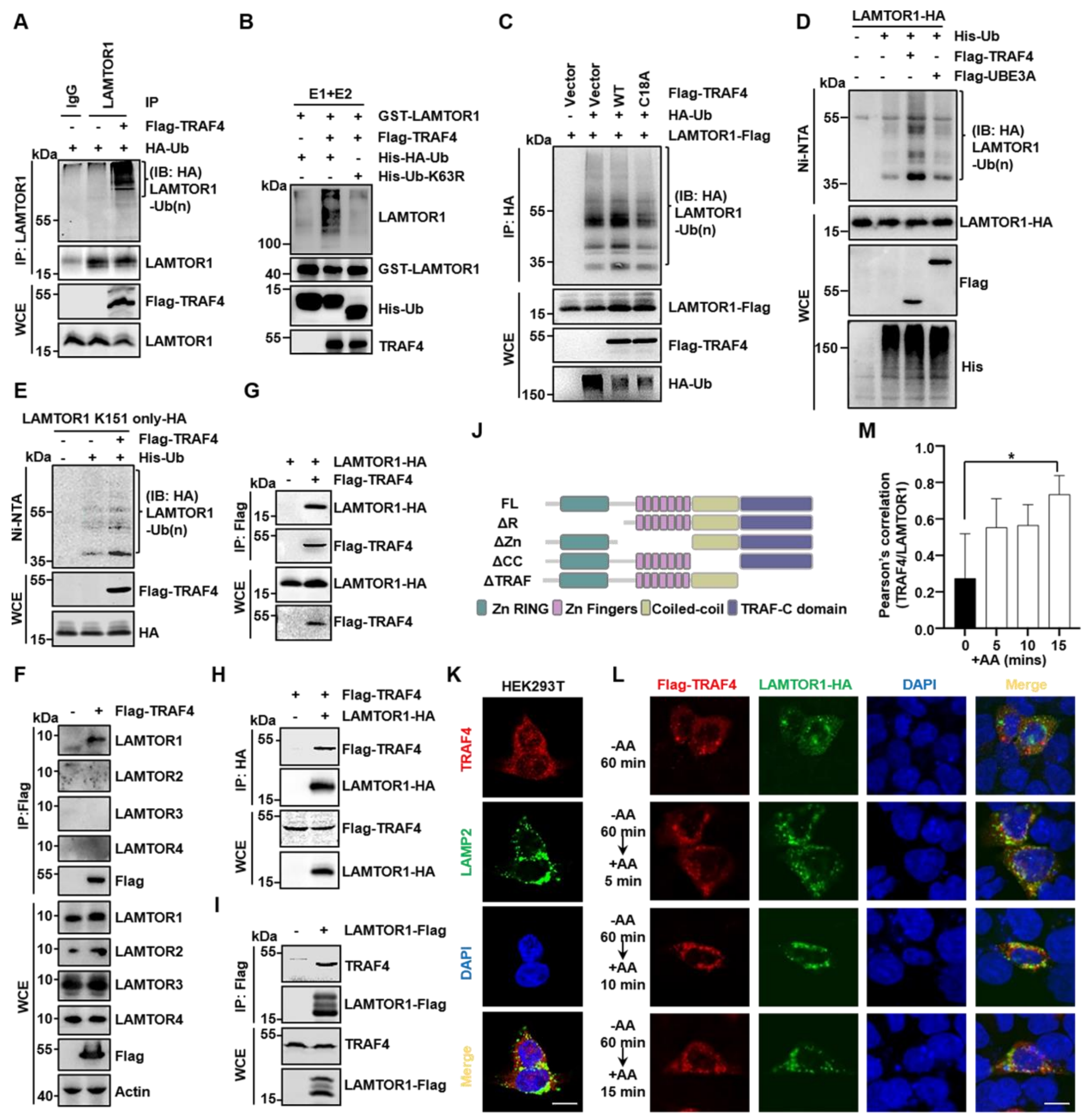

Figure S2. TRAF4 as an E3 ligase of LAMTOR1

A) TRAF4 promoted the ubiquitination of endogenous LAMTOR1. HEK293T cells were transfected with indicated plasmids. A ubiquitination assay was conducted by Ni-NTA pull down under denaturing condition following Western blotting. B) In vitro ubiquitination indicated that TRAF4 directly promote the K63 polyubiquitination of LAMTOR1. In vitro ubiquitination assays were performed with Flag-TRAF4 and GST-LAMTOR1, E1 (UBE1), E2 (UbcH6), ubiquitin and Ub-K63R. C) The ubiquitination of LAMTOR1 depended on ubiquitination enzyme activity. TRAF4-C18A is a mutant with loss of kinase activity. D) TRAF4 but not UBE3A promoted ubiquitination of LAMTOR1 in HEK293T cells. E) TRAF4 promoted the ubiquitination of LAMTOR1-

K151 only mutant. F) Exogenous over expression of Flag-TRAF4 interacted with endogenous LAMTOR1 in HEK293T cells, but not the other components of Ragulator. G-H) LAMTOR1 binds to TRAF4. LAMTOR1-HA and FLAG-TRAF4 were cotransfected into HEK293T cells, and cells were lysed. TRAF4 was immunoprecipitated by anti-FLAG antibody. Coimmunoprecipitated LAMTOR1 was detected by anti-HA-antibody. I) Exogenous over expression of LAMTOR1-Flag interacted with endogenous TRAF4 in HEK293T cells. J) The schematic diagram of TRAF4. K) TRAF4 localizes on the surface of lysosome. Immunofluorescent staining for endogenous TRAF4 (red) and lysosome marker LMAP2 (green). Scale bar, 10 µm. L) Exogenous overexpression of FLAG-TRAF4 and LAMTOR1-HA in HEK293T cells. Immunofluorescent staining for FLAG-TRAF4 (red) and LAMTOR1-HA (green). Scale bar, 10 µm. M) Pearson's correlation quantifies exogenous TRAF4/LAMTOR1 colocalization, mean ± SD ($n \geq 3$), two-tailed Student's t test, $*P < 0.05$.

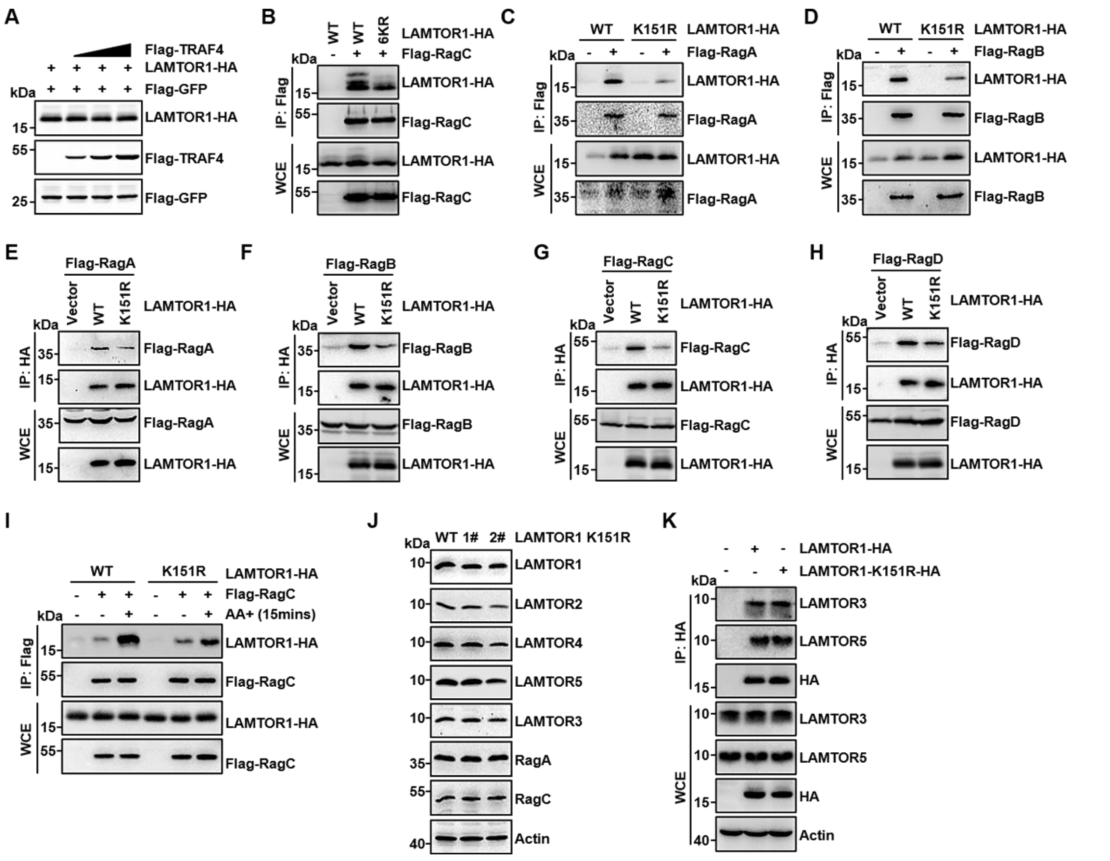

Figure S3. The ubiquitination of LAMTOR1 promotes its interaction with GTPases

A) TRAF4 did not affect the protein stability of LAMTOR1. Both TRAF4 and LAMTOR1 were overexpressed in HEK293T cells, and the protein stability of LAMTOR1 was detected by Western Blotting. B) LAMTOR1-6KR mutation decreased its interaction with RagC. C-H) The interaction between RagA, RagB, RagC, RagD and LAMTOR1-K151R was decreased comparing to LAMTOR1-WT. I) The interaction of LAMTOR1 K151R with RagC decreased comparing with LAMTOR1-WT by amino acids restimulation. J) The expressions of five components of Ragulator had no discrimination in HEK293T cell line with LAMTOR1-K151R mutation. K) Exogenous overexpression of LAMTOR1-K151R-HA did not affect the interaction between LAMTOR1 and the other components of Ragulator compare with LAMTOR1-HA-WT.

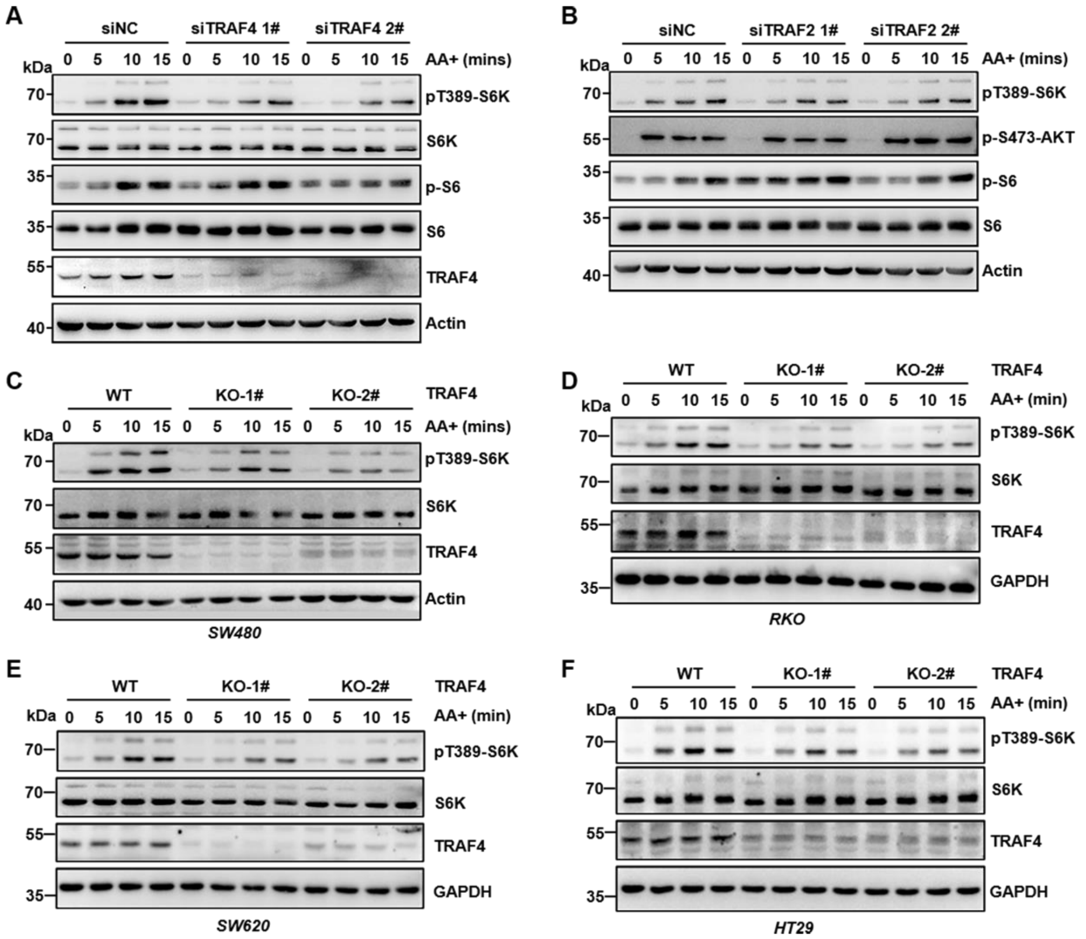

Figure S4. TRAF4 promoted mTORC1 activation

A-B) Knockdown of TRAF4 but not TRAF2 reduces amino acid–dependent mTORC1 signaling in HEK293T cells. HEK293T cells were treated with nonspecific control siRNA (siNC), TRAF4-targeting siRNA (siTRAF4) or siTRAF2. Cells were starved of amino acids for 60 min and re-stimulated with amino acids for 5min, 10min, 15min. C-F) The activation of mTORC1 signaling pathway was detected in colorectal cancer cells SW480, RKO, SW620 and HT29.

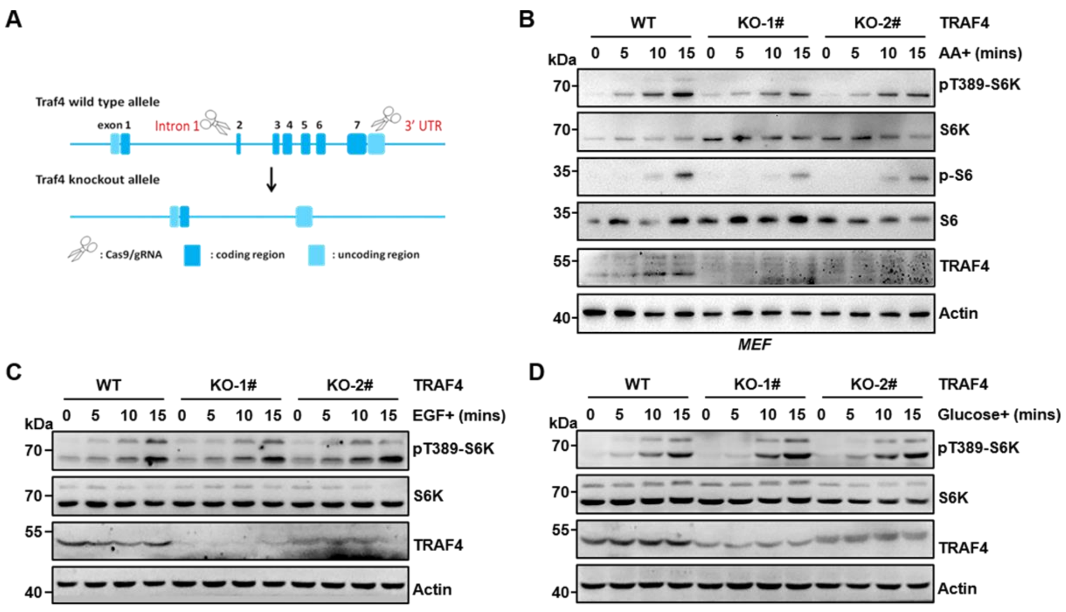

Figure S5. TRAF4 deficiency inhibited the amino acid-induced mTORC1 activation

A-B) The activation of mTORC1 signaling pathway was detected in *TRAF4*$^{-/-}$ MEF cell lines. MEF, Mouse Embryonic Fibroblast. C-D) The glucose and EGF signaling had no effect on the activity of mTORC1 in HEK293T cells.

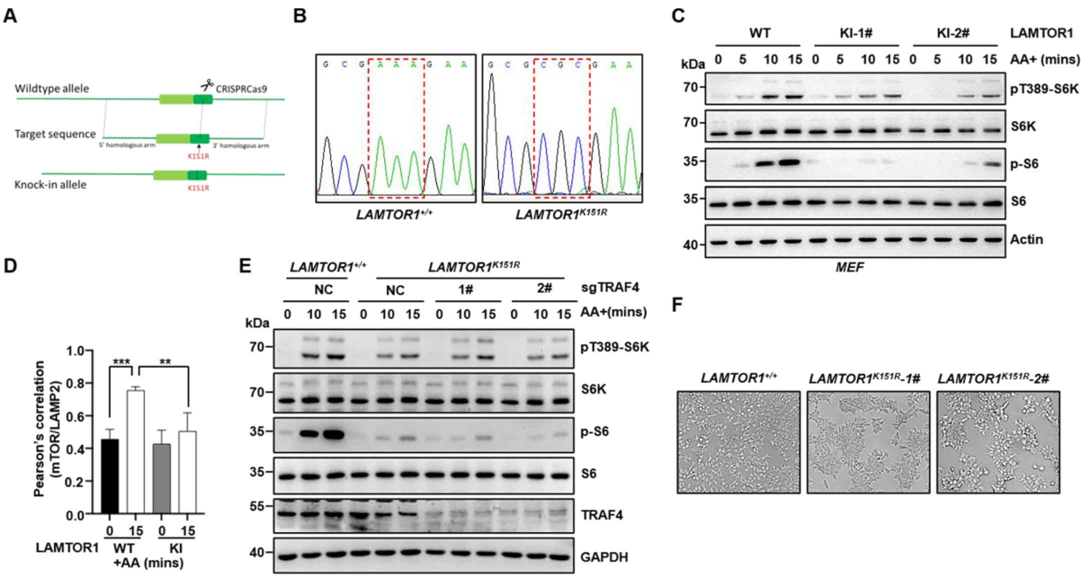

Figure S6. LAMTOR1 ubiquitination promoted mTORC1 activation

A-C) The activation of mTORC1 signaling pathway was detected in *LAMTOR1*^{K151R} knockin MEF cell lines. D) Pearson's correlation quantifies endogenous mTOR/LAMP1 colocalization, mean ± SD ($n \geq 5$), two-tailed Student's t test, $^{**}P < 0.01$, $^{***}P < 0.001$. Quantification of co-localization of mTORC1 and LAMP2 with amino acid stimulation. E) The activation of mTORC1 signaling pathway was decreased in TRAF4 deficiency HEK293T K151R cells. F) Phase-contrast images of growing *LAMTOR1*$^{+/+}$ (left) and *LAMTOR1*^{K151R} (right) cells.

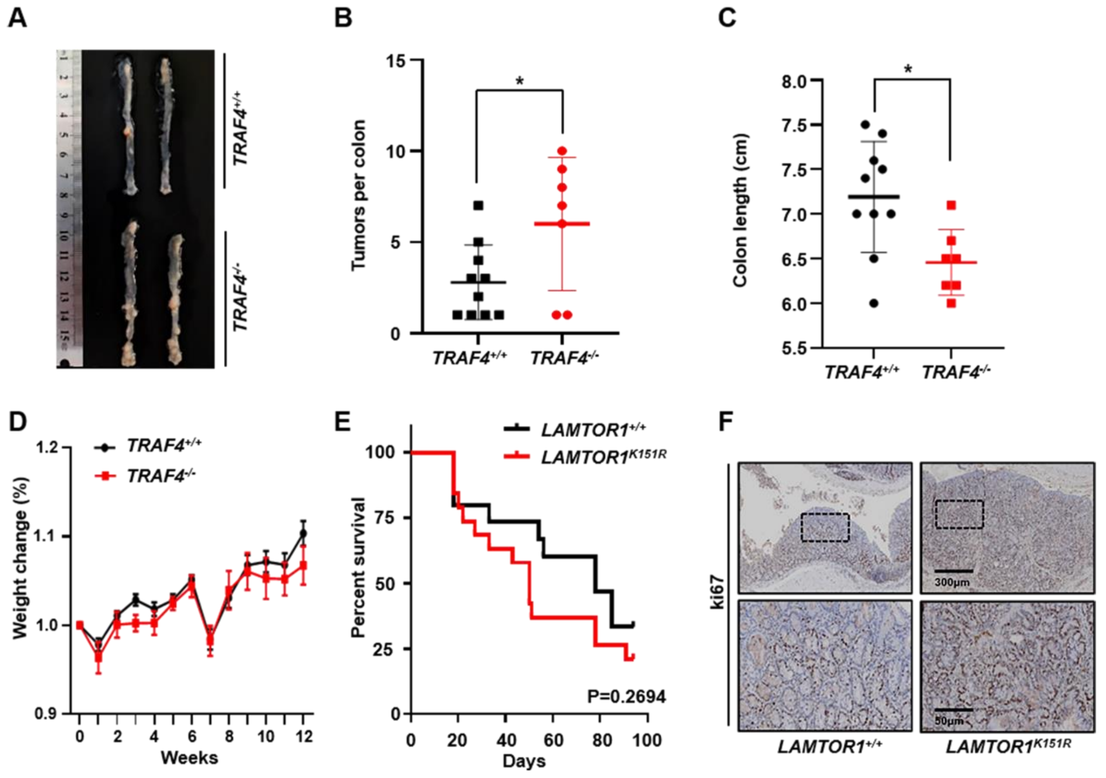

Figure S7. The TRAF4 knock-out mice inhibit colon cancer

A) Representative images of colon tumors in TRAF4 WT(*TRAF4*$^{+/+}$) (n=10) and TRAF4 KO(*TRAF4*$^{-/-}$) mice (n=7) after 12 weeks of AOM/DSS induction. *TRAF4* $^{-/-}$ mice using an AOM/DSS inflammation-induced colon cancer model consistent with LAMTOR1 K151R mice. B) Number of polyps from *TRAF4*$^{+/+}$ (n=10) and *TRAF4*$^{-/-}$ (n=7) mice treated with AOM/DSS. Data were analyzed by Student's t-test. P< 0.05 was considered statistically significant. *P< 0.05. C) Colorectal length from *TRAF4*$^{+/+}$ (n=10) and *TRAF4*$^{-/-}$ (n=7) mice treated with AOM/DSS. Data were analyzed by Student's t-test. P< 0.05 was considered statistically significant. *P< 0.05. D) Growth curve of TRAF4 WT(*TRAF4*$^{+/+}$) (n=10) and TRAF4 KO(*TRAF4*$^{-/-}$) (n=7) mice in 12 weeks. E) Kaplan-Meier curve of *LAMTOR1*$^{+/+}$ (n=15) and *LAMTOR1*^{K151R} (n=19) mice treated with AOM/DSS, log-rank (Mantel-Cox) text, P < 0.05 was considered statistically significant, ns, not significant. F) Representative immunohistochemical ki67 staining of AOM/DSS colon cancer model.
